# Evaluating XAI techniques under class imbalance using CPRD data

**DOI:** 10.3389/frai.2025.1682919

**Published:** 2025-11-13

**Authors:** Teena Rai, Jun He, Jaspreet Kaur, Yuan Shen, Mufti Mahmud, David J. Brown, Emma O'Dowd, David Baldwin

**Affiliations:** 1 Department of Computer Science, Nottingham Trent University, Nottingham, United Kingdom; 2 Division of Epidemiology and Public Health, University of Nottingham, Nottingham, United Kingdom; 3 Department of Information and Computer Science, King Fahd University of Petroleum and Minerals, Dhahran, Saudi Arabia

**Keywords:** eXplainable AI, LIME, SHAP, PDP, class imbalance, CPRD, evaluation, consistency

## Abstract

**Introduction:**

The need for eXplainable Artificial Intelligence (XAI) in healthcare is more critical than ever, especially as regulatory frameworks such as the European Union Artificial Intelligence (EU AI) Act mandate transparency in clinical decision support systems. *Post hoc* XAI techniques such as Local Interpretable Model-Agnostic Explanations (LIME), SHapley Additive exPlanations (SHAP) and Partial Dependence Plots (PDPs) are widely used to interpret Machine Learning (ML) models for disease risk prediction, particularly in tabular Electronic Health Record (EHR) data. However, their reliability under real-world scenarios is not fully understood. Class imbalance is a common challenge in many real-world datasets, but it is rarely accounted for when evaluating the reliability and consistency of XAI techniques.

**Methods:**

In this study, we design a comparative evaluation framework to assess the impact of class imbalance on the consistency of model explanations generated by LIME, SHAP, and PDPs. Using UK primary care data from the Clinical Practice Research Datalink (CPRD), we train three ML models: XGBoost (XGB), Random Forest (RF), and Multi-layer Perceptron (MLP), to predict lung cancer risk and evaluate how interpretability is affected under class imbalance when compared against a balanced dataset. To our knowledge, this is the first study to evaluate explanation consistency under class imbalance across multiple models and interpretation methods using real-world clinical data.

**Results:**

Our main finding is that class imbalance in the training data can significantly affect the reliability and consistency of LIME and SHAP explanations when evaluated against models trained on balanced data. To explain these empirical findings, we also present a theoretical analysis of LIME and SHAP to understand why explanations change under different class distributions. It is also found that PDPs exhibit noticeable variation between models trained on imbalanced and balanced datasets with respect to clinically relevant features for predicting lung cancer risk.

**Discussion:**

These findings highlight a critical vulnerability in current XAI techniques, i.e., their interpretability are significantly affected under skewed class distributions, which is common in medical data and emphasises the importance of consistent model explanations for trustworthy ML deployment in healthcare.

## 1 Introduction

Local Interpretable Model-Agnostic Explanations (LIME) and SHapley Additive exPlanations (SHAP) are among the most widely adopted techniques for interpreting machine learning (ML) models in disease risk prediction tasks (Alabi et al., 2023; Fu et al., 2024; Rai et al., 2025). Despite their popularity, there is a lack of rigorous evaluation of their reliability in clinical contexts, particularly under conditions of severe class imbalance, which are common in real-world healthcare datasets (Hassanzadeh et al., 2023; Rahman and Davis, 2013; Xu et al., 2020). Diseases such as lung cancer illustrate this challenge because the prevalence is low in the general population, but they are associated with a high mortality rate (Navani et al., 2022) largely due to late-stage diagnosis.

While some recent studies on diabetes prediction has introduced interpretability metrics, they were not directly integrated into their model training and evaluation pipeline (Ahmed et al., 2024). Furthermore, studies addressing class imbalance typically apply resampling strategies such as Synthetic Minority Oversampling Technique (SMOTE), oversampling, and undersampling prior to interpreting models with LIME or SHAP (Advithi and Umadevi, 2024; Kokkotis et al., 2022). However, very few have critically examined how these resampling strategies influence the trustworthiness or consistency of model explanations. For example, rule-based model research has shown that resampling techniques can substantially alter feature importance rankings (Gao et al., 2022), and similar findings have been reported for global explanation techniques like Partial Dependence and Accumulated Local Effects profiles (Stando et al., 2024).

According to (Molnar 2025), stability refers to the extent to which explanations remain similar for similar input instances for the same model. A recent study has explored explanation stability under class imbalance, demonstrating that balanced datasets often yield more stable explanations (Chen et al., 2024). Background data in SHAP refers to the dataset used to marginalize over “absent” features when estimating Shapley values. Studies have shown that the background data used in SHAP can influence explanation stability (Ihalapathirana et al., 2024), with larger background datasets generally producing more stable results (Yuan et al., 2022). In this study, we define consistency as the degree to which explanation outputs given by LIME and SHAP remain similar across models trained under different class distributions for the same predictive task (Molnar, 2025). While a study (Chen et al., 2024) examines explanation stability by analyzing variability across multiple training runs under class imbalance, our study takes a different approach by systematically evaluating how explanation consistency varies across models trained on datasets with different class distributions, using a balanced model as a reference.

The main contributions of the study are as listed in the following:

1. We design a comparative framework using Jaccard similarity index and Rank Agreement to evaluate the consistency of LIME and SHAP explanations under class imbalance in predicting lung cancer risk from Clinical Practice Research Datalink (CPRD) data. To our knowledge, no prior research has comprehensively investigated how the consistency and reliability of LIME and SHAP explanations are affected by class imbalance relative to a balanced model, using real-world clinical data.
2. Changes in explanation rankings across different class distributions are assessed, and their relationship with model performance is examined to identify potential trade-offs between model performance and interpretability.
3. We apply Partial Dependence Plots (PDPs) as part of our evaluation framework to assess how the model's learning of clinically relevant features for predicting lung cancer risk changes across balanced and imbalanced datasets.
4. We analyse the internal mechanisms of LIME and SHAP to explain why class imbalance influences explanation consistency, providing a theoretical context for observed empirical patterns.

This study is a significant expansion of our accepted short study presented at the *4th International Workshop on Explainable Artificial Intelligence in Healthcare*, held at the *23rd International Conference on Artificial Intelligence in Medicine*. The workshop study focused only on the first contribution, that is, evaluating the consistency of LIME and SHAP under class imbalance. The current study adds several new components: (i) an analysis of the relationship between class imbalance, model performance and interpretability, (ii) the use of PDPs to evaluate model behavior on clinically relevant features in predicting lung cancer risk, and (iii) a deeper theoretical and empirical investigation of how the consistency of explanations based on LIME and SHAP varies under class imbalance and how this impacts interpretability.

The structure of the study is as follows. Section 2 outlines the methodology employed to evaluate the consistency of LIME, SHAP, and PDPs under varying levels of class imbalance using CPRD data. Moreover, the theoretical foundations of LIME and SHAP are discussed in detail to explain why explanation consistency is affected under different class distributions. Section 3 presents the results from evaluating the machine learning (ML) models, analyzing LIME and SHAP consistency under class imbalance and PDP visualizations across models trained under different class distributions. Furthermore, an empirical illustration of variation in the explanations provided by LIME and SHAP is presented. Section 4 discusses the need for systematic evaluation of eXplainable Artificial Intelligence (XAI) techniques. Finally, Section 5 summarizes the key findings and implications of the study.

## 2 Methodology

### 2.1 Data

In this study, CPRD data (Herrett et al., 2015), a comprehensive UK-based primary care database containing anonymised patient records from general practices across the UK, was used. The primary outcome of interest was the diagnosis of lung cancer. By looking at data between 1 January 2014 and 1 January 2020, our cohort consisted of 1,390,070 non-lung cancer cases and 8,412 lung cancer cases, resulting in a highly imbalanced dataset with lung cancer cases comprising approximately 0.6 % of the total. The predictive task was formulated as a binary classification problem. The features used during model training were *age, smoking status and intensity, history of bronchiectasis, history of cerebrovascular disease, history of chronic kidney disease, Chronic Obstructive Pulmonary Disease (COPD)/emphysema, diabetes with end-stage complications, diabetes without end-stage complications, family history of cancer, family history of lung cancer, idiopathic fibrosis, lower respiratory tract infections*, body mass index *(BMI) status, peptic ulcer, alcohol status, peripheral vascular disease, radio therapy*, and personal history of *breast cancer, bladder cancer, head and neck cancer* and *thyroid cancer*. Finally, symptoms reported within the year preceding the 2-year period before follow-up—*dyspnoea, haemoptysis, cough, sputum production, back pain*, and any *blood test performed* were also included.

### 2.2 Model training and evaluation

The data was split into 80% for training and 20% for testing, with stratification applied to ensure the proportion of lung cancer cases was preserved in both sets. Hence, the training data had 6,730 lung cancer cases and 1,112,055 non-lung cancer cases. To investigate the effect of varying degrees of class imbalance on LIME and SHAP explanations, several training subsets of identical sample size were randomly selected from the original training data. Each subset consisted of different proportions of lung cancer cases: 40%, 30%, 20%, 10%, 5%, and 1%, and the original (highly imbalanced) distribution. A balanced dataset with 50% lung cancer cases and 50% non-lung cancer cases served as the reference which was obtained by using random undersampling. A balanced model was used as the reference because we found that most studies used some form of balancing techniques, such as SMOTE, undersampling, and oversampling, to overcome the class imbalance problem in their dataset as part of their data preprocessing before model development (Abdoh et al., 2018; Bhavani and Govardhan, 2023; Hsu et al., 2015). We selected undersampling because the dataset contained a sufficient number of the minority (lung cancer cases) class to construct balanced data without the need to generate synthetic samples (with SMOTE) or duplicate existing cases (with oversampling) (Hasanin and Khoshgoftaar, 2018). More details with the number of lung cancer and non-lung cancer cases for the different class distributions used during model training can be found in Table 1.

**Table 1:** Number of lung cancer and non-lung cancer cases in the training data under varying percentage of lung cancer cases.

**Percentage of lung cancer cases**	**Number of lung cancer cases**	**Number of non-lung cancer cases**
Balanced	6,730	6,730
40%	5,384	8,076
30%	4,038	9,422
20%	2,692	10,768
10%	1,346	12,114
5%	673	12,787
1%	135	13,325
Original	82	13,378

Three ML models: eXtreme Gradient Boosting (XGB), Random Forest (RF) and Multi Layer Perception (MLP) were trained on each of these subsets in Table 1. A balanced testing set with 1,682 lung cancer cases and 1,682 non-lung cancer cases was used for model evaluation. Heatmaps of area under the curve (AUC), sensitivity, and specificity across the different training subsets were used to assess whether explanation consistency is linked to changes in model performance. Models were implemented in Python using scikit-learn and XGBoost (Chen and Guestrin, 2016) libraries. For MLP, default hyperparameters were used. For RF and XGB, the *max_depth* parameter was set at 5, while other parameters were left at their defaults. No hyperparameter optimisation was performed because the primary aim of this study was to compare explanation consistency across models rather than maximize predictive performance.

### 2.3 Metrics for measuring consistency

We designed a comparative framework using two metrics to systematically assess explanation consistency under varying class distributions. First, LIME and SHAP explanations for predicting lung cancer risk were generated for the same 100 randomly selected test instances across all models trained with different class distributions as described in Section 2.2. Second, to capture whether the set of top-10 ranked features remains consistent across models trained under different class distributions, we compute the Jaccard similarity index between the top-10 feature rankings of each model and those of the balanced reference model. Third, to assess whether the ordering of these features is preserved, we calculate the Rank Agreement which measures the concordance in ranking positions across models. Moreover, a separate class-wise analysis was also performed to see the trends observed in explanation consistency for the minority (lung cancer cases) and majority (non-lung cancer cases) classes. LIME and SHAP explanations for 100 lung cancer and 100 non-lung cancer cases were randomly selected from the test dataset across all models. For each instance, explanations from the imbalanced models were compared with those of their respective balanced models. The mean Jaccard similarity and Rank Agreement across the 100 instances were reported with error bars indicating the variability in the explanation consistency of the minority and majority classes separately. These metrics together allow us to evaluate not only which features are deemed important across different models but also whether their relative importance is consistent. This approach enables a more rigorous assessment of explanation consistency beyond simple overlap. The metrics have been defined in detail below.

1. Given two explanations *E*_*a*_ and *E*_*b*_, the Jaccard similarity index is defined as (Verma and Aggarwal, 2020):

$$\begin{array}{l} \text{Jaccard Similarity Index}=\frac{\left|\text{Top}\left(E_{a},k\right)\cap \text{Top}\left(E_{b},k\right)\right|}{\left|\text{Top}\left(E_{a},k\right)\cup \text{Top}\left(E_{b},k\right)\right|} \end{array}$$ (1)

where Top(*E, k*) denotes the set of top *k* features of explanation *E* and |.| denotes the cardinality of the set.

2. Rank Agreement as discussed in a previous study (Krishna et al., 2022) measures how consistently the top *k* features are ordered across different explanations *E*_*a*_ and *E*_*b*_. However, their approach only considers the top *k* features in the denominator when calculating Rank Agreement. In contrast, our method differs by considering the union of the top *k* features from the explanations *E*_*a*_ and *E*_*b*_, similar to the Jaccard similarity index. The rank agreement is defined as:

$$\begin{array}{l} \frac{\left|\underset{s\in S}{⋃}\left\{s|\left(s\in \text{Top}\left(E_{a},k\right)\cap \text{Top}\left(E_{b},k\right)\right)\text{and rank}\left(E_{a},s\right)=\text{rank}\left(E_{b},s\right)\right\}\right|}{\left|\text{Top}\left(E_{a},k\right)\cup \text{Top}\left(E_{b},k\right)\right|} \end{array}$$ (2)

where *S* is the set of features in the data, Top(*E, k*) and |.| are defined as above and rank(*E, s*) denotes the position or rank of the feature *s* according to the explanation *E*.

We use an example to illustrate how Rank Agreement is calculated. For simplicity, we consider two ordered rankings, each with five elements: Ranking 1 = {*a*, *b*, *c*, *d*, *e*} and Ranking 2 = {*a*, *f*, *g*, *d*, *e*}. Three elements *a*, *d*, and *e* appear in both rankings, and they also maintain the same relative order in both sets. The total number of distinct elements across both rankings (the union) is 7. Therefore, the rank agreement using Equation 2 is 3/7.

### 2.4 Partial dependence plots

To investigate how class imbalance influences model behavior, we incorporated PDPs as part of our evaluation framework. We focused on two clinically important features for predicting lung cancer risk: *age* and *smoking status and intensity* provided by (Callender et al. 2023) and (Guo et al. 2022), where *age* is a continuous variable and *smoking status and intensity* is a categorical variable with nine levels (Ex Unknown, Ex Light, Ex Moderate, Ex Heavy, Current Unknown, Current Light, Current Moderate, Current Heavy, and Missing). For models trained on both the balanced dataset and the original highly imbalanced data, we generated PDPs to examine how predicted risk varies with respect to these features. This enabled a systematic comparison of how class distribution in training data affects the model's sensitivity to learning of clinically meaningful features.

PDPs show the marginal effect a feature has on the predicted outcome of an ML model. Let *X* be the dataset, *S* be the set of features we are interested in knowing the effects on the predicted outcome, and *C* denote the set of features which are not in *S*. Hence, *x*_*S*_ are the features we are interested in plotting the partial dependence function and *X*_*C*_ are the remaining features in the model $\tilde{f}$. The feature vectors *x*_*S*_ and *X*_*C*_ make the dataset *X*. The partial dependence function is defined as


$$\begin{array}{l} \tilde{f_{S}}\left(x_{S}\right)={𝔼}_{X_{C}}\left[\tilde{f}\left(x_{S},X_{C}\right)\right]=\int \tilde{f}\left(x_{S},X_{C}\right)d\mathbb{P}\left(X_{C}\right) \end{array}$$ (3)

where E_*X*_*C*__ represents the expected value of the function f~(x_*S*_, X_*C*_) with respect to the probability distribution of the features X_*C*_, denoted by P(X_*C*_).

From Equation 3, we can see that PDP works by marginalizing the output of the ML model over the distribution of features in *C* so that we can see the relationship between the function in set *S* and the predicted outcome.

Let *n* be the number of instances in the dataset. The partial function $\tilde{f_{S}}$ is calculated by averaging over the training data.


$$\begin{array}{l} \tilde{f_{S}}\left(x_{S}\right)=\frac{1}{n}\overset{n}{\underset{i=1}{\sum }}\tilde{f}\left(x_{S},x_{C}^{\left(i\right)}\right) \end{array}$$ (4)

In our case, where we are interested in binary classification and the feature (*age*) is continuous, the PDPs display the probability of developing lung cancer for different values of the features in *S*, holding the features in *C* fixed. For categorical features (*smoking status and intensity*), we get a PDP estimate by forcing all data instances to have the same category. These plots were compared between models trained on the balanced dataset and those trained on the original, highly imbalanced data, allowing visual inspection of how the learned relationships between features and predictions differed.

### 2.5 LIME and imbalanced data

In this section, we examine how LIME works to explain why its explanations change under different class distributions.

LIME (Ribeiro et al., 2016) approximates a black box model *f* with a simple interpretable model *g*∈*G* locally around an instance *x*:


$$\begin{array}{l} \text{explanation}\left(x\right)=\text{arg}\underset{g\in G}{\min}\underset{x^{\prime }\in Z}{\sum }\pi_{x}\left(x^{\prime }\right){\left(f\left(x^{\prime }\right)-g\left(x^{\prime }\right)\right)}^{2}+\text{Ω}\left(g\right), \end{array}$$ (5)

where *Z* is a set of perturbed samples around *x*, $\pi_{x}\left(x^{\prime }\right)$ is a proximity measure between *x* and *x*′ that assigns higher weight to samples closer to *x*, *f*(*x*′) is the black box model's prediction on perturbed sample *x*′∈*Z*, *g*(*x*′) is the surrogate (or interpretable) model's prediction and Ω(*g*) is a regularization term controlling the complexity of the surrogate model.

For a model trained on imbalanced data with a majority negative class, most predictions on the perturbed sample *x*′∈*Z* will be of the negative class, that is, *f*(*x*′) = 0 in many cases. This skews the local neighborhood toward a single class, and the surrogate model *g*, which is trained on these targets, learns the decision boundary of the negative class rather than the local points around *x*. The explanation may no longer reflect what influences the positive prediction for *x*.

Suppose the surrogate model *g* is a linear model:


$$\begin{array}{l} g\left(x^{\prime }\right)=\beta_{0}+\overset{d}{\underset{j=1}{\sum }}\beta_{i}x_{j}^{\prime } \end{array}$$ (6)

The optimisation problem in Equation 5 becomes


$$\begin{array}{l} \text{arg}\underset{\beta }{\min}\underset{x^{\prime }\in Z}{\sum }\pi_{x}\left(x^{\prime }\right){\left(f\left(x^{\prime }\right)-\beta_{0}-\overset{d}{\underset{j=1}{\sum }}\beta_{i}x_{j}^{\prime }\right)}^{2} \end{array}$$ (7)

Under imbalanced data where most *f*(*x*′) = 0, *g*(*x*′) becomes biased toward approximating the negative class. The learned coefficients β_*j*_ then no longer represent the local contribution of each feature to the prediction at *x*, causing different explanations when compared across models trained on balanced datasets.

### 2.6 SHAP and imbalanced data

In this section, we analyse SHAP's underlying mechanism to explain changes in its explanations across different training class distributions.

SHAP (Lundberg and Lee, 2017) values are grounded in cooperative game theory, originally proposed by Lloyd Shapley in 1953. In this framework, the model prediction represents the “payout” of the game, and input features act as “players” contributing to that payout. SHAP values aim to fairly distribute the model's output among the features based on their marginal contributions.

Let *p* be the number of features in dataset *X*∈ℝ^*p*^ and let *S*⊆{1, …, *p*} be a set of features. Let us denote the complement of S as *S*^*C*^ = {1, …, *p*}\*S*, that is, the set of features not in *S* and *i*∈{1, …, *p*} be the features of interest.

The Shapley value for a feature *i* and data instance *x*^(*j*)^ is (Molnar, 2025):


$$\begin{array}{l} \phi_{i}\left(f,x^{\left(j\right)}\right)=\underset{S\subseteq \left\{1,\ldots ,p\right\}\backslash \left\{i\right\}}{\sum }\frac{|S|!\left(p-|S|-1\right)!}{p!}\left[\left(v\left(S\cup \left\{i\right\}\right)-v\left(S\right)\right)\right], \end{array}$$ (8)

where *v*(*S*) is the model's expected output when only the features in *S* are known. This value function is formally defined as:


$$v(S)={\text{𝔼}}_{X_{S^{C}}}[f(X_{S},X_{S^{C}}],$$ (9)

which can be rewritten as an integral over the marginal distribution:


$$\begin{array}{l} v\left(S\right)=\int f\left(X_{s}\cup X_{S^{C}}\right)\mathbb{P}\left(X_{S^{C}}|X_{S}\right)dX_{S^{C}} \end{array}$$ (10)

Here, $\mathbb{P}\left(X_{S^{C}}|X_{S}\right)$ reflects the conditional distribution of the unknown features *S*^*C*^ given the known ones *S*. The conditional distribution and therefore the value function depends directly on the overall data distribution ℙ(*X*).

In imbalanced datasets, ℙ(*X*) is heavily skewed toward the majority class. However, when the model is trained on balanced data, the training distribution is changed to ℙ_balanced_(*X*), where:


$$\begin{array}{l} {\mathbb{P}}_{\text{balanced}}\left(Y=1\right)\approx {\mathbb{P}}_{\text{balanced}}\left(Y=0\right) \end{array}$$ (11)

This changes both the learned model *f* and the conditional distribution ${\mathbb{P}}_{\text{balanced}}\left(X_{S^{C}}|X_{S}\right)|$ in Equation 10. Therefore, the SHAP value function *v*(*S*) is computed over a different distribution, that is,


$$\begin{array}{l} v_{\text{imbalanced}}\left(S\right)\neq v_{\text{balanced}}\left(S\right)\;\Rightarrow \;\phi_{i}^{imbalanced}\neq \phi_{i}^{balanced} \end{array}$$ (12)

Moreover, when models are trained on imbalanced data, the prediction for the minority class shrinks, which in turn shrinks the value function *v*(*S*) and, in turn, shrinks (Equation 8). We will see an empirical example of this in Section 3.2.1.

Because global feature importance scores are derived from mean absolute SHAP values across the dataset:


$$\begin{array}{l} I_{i}=\frac{1}{n}\overset{n}{\underset{j=1}{\sum }}|\phi_{i}\left(f,x^{\left(j\right)}\right)|, \end{array}$$

it follows that a shift in the underlying distribution ℙ(*X*) will influence the SHAP values ϕ_*i*_ and thus resulting importance scores *I*_*i*_. This results in different feature rankings when comparing models trained on imbalanced compared to balanced datasets.

## 3 Results

### 3.1 Evaluation of model performance under class imbalance

**Figure 1:**
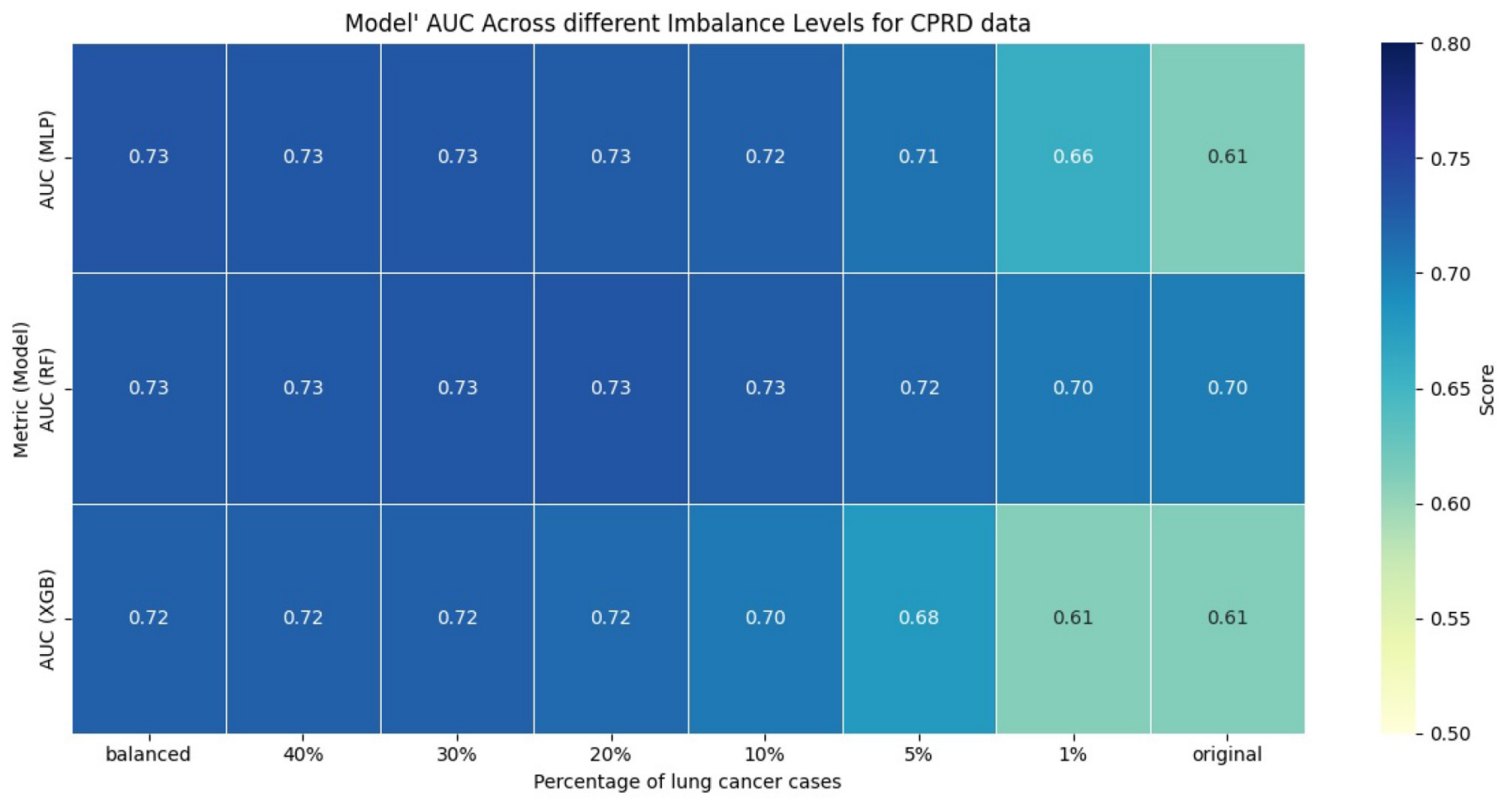
AUC of models across different imbalance levels.

**Figure 2:**
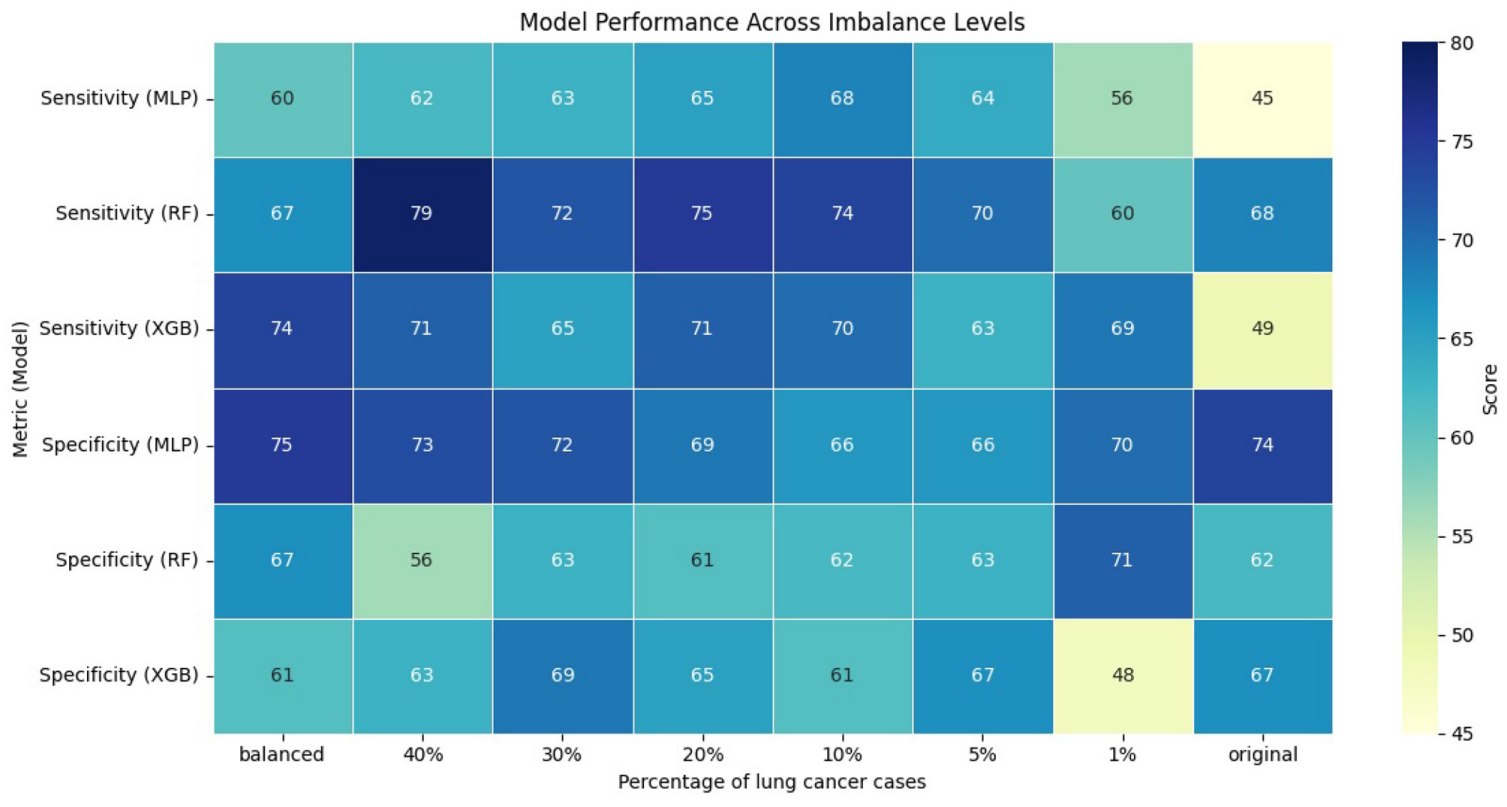
Performance of models across different imbalance levels.

The performance of the models was evaluated using AUC, sensitivity, and specificity. We computed sensitivity and specificity for all models at optimal thresholds, with different levels of class imbalance, using Youden's *J* index (Schisterman et al., 2008). Figures 1, 2 show the model's performance of MLP, RF, and XGB trained across different levels of class imbalance. The highest AUC is achieved for RF and MLP when trained on balanced data with 40%, 30%, and 20% lung cancer cases. The AUC is also consistently high for RF at all levels of class imbalance. The AUC of both MLP and XGB starts dropping as the lung cancer cases drop to 5% and goes down further when trained under the original imbalanced class distribution. Hence, the AUC of the models dropped as the level of class imbalance increased. Similarly, RF achieved the highest sensitivity when trained with 40% lung cancer cases, with sensitivity remaining comparable across models trained under different levels of class imbalance. Both XGB and MLP maintained a good trade-off between sensitivity and specificity across all imbalance levels.

To evaluate whether the observed differences in AUC, sensitivity, and specificity were statistically significant, we compared each imbalanced model with the balanced reference model using the Mann-Whitney U test. We applied bootstrapping on the test dataset to generate 100 bootstrap samples, from which we obtained distributions of model performance for each metric. The null hypothesis stated that there was no difference in performance between balanced and imbalanced models, while the alternative hypothesis stated that there was a difference. Across all comparisons and metrics, the *p*-value was less than 0.01, indicating statistically significant differences in performance of imbalanced models compared to the balanced reference model.

### 3.2 Evaluation of LIME and SHAP under class imbalance

#### 3.2.1 Evaluation using consistency metrics

**Figure 3:**
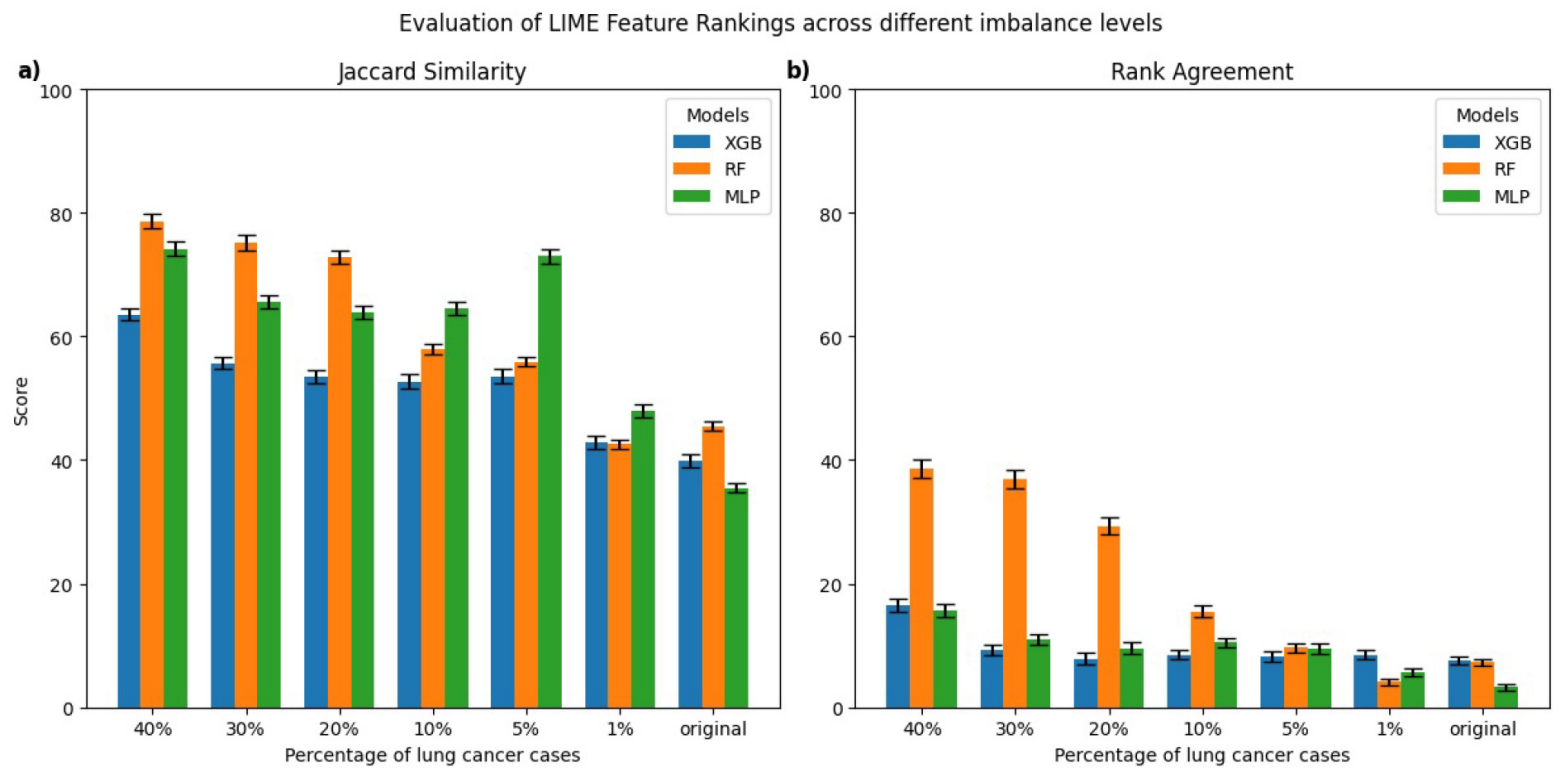
Comparison of top-10 feature rankings generated by LIME across varying percentages of lung cancer cases, evaluated against the balanced model using **(a)** Jaccard Similarity Index and **(b)** Rank Agreement. All error bars shown in the figures throughout the study represent the standard error.

**Figure 4:**
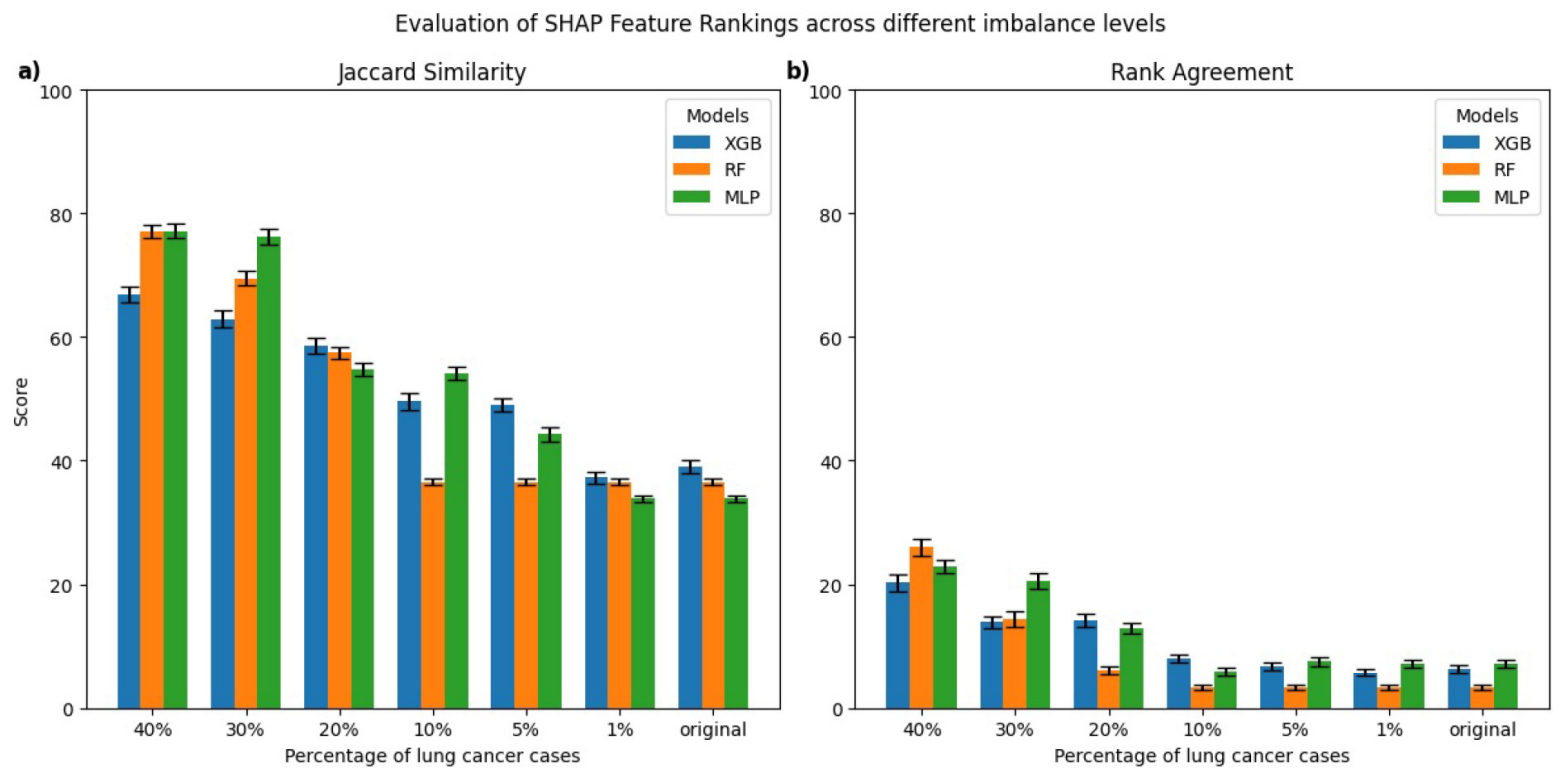
Comparison of top-10 feature rankings generated by SHAP across varying percentages of lung cancer cases, evaluated against the balanced model using **(a)** Jaccard Similarity Index and **(b)** Rank Agreement.

Figures 3, 4 present the evaluation of top-10 feature rankings given by LIME and SHAP across models trained with varying percentages of lung cancer cases relative to a reference balanced model using Jaccard similarity index and Rank Agreement.

For LIME, the highest Jaccard similarity with respect to the balanced model was observed in the RF model trained on 40% lung cancer cases with a gradual decline in Jaccard similarity as the class imbalance increased (Figure 3a). A similar trend was observed for XGB and MLP, where models trained on 40% lung cancer cases showed relatively high Jaccard similarity, followed by a gradual decline as class imbalance increased and reached its lowest values for models trained on the original imbalanced distribution. In terms of rank agreement, similar patterns were observed with rank agreement highest for models trained on 40% lung cancer cases and decreasing as the percentage of cases declined (Figure 3b). Moreover, for all models, the rank agreement between the balanced and original imbalanced models remained below 10%, indicating minimal overlap in the most important features identified by LIME.

For SHAP, the MLP models trained on 30%–40% lung cancer cases showed the highest Jaccard similarity with the balanced reference (Figure 4a). As the degree of imbalance increased, the Jaccard similarity gradually declined, mirroring similar patterns observed with LIME. RF and XGB models showed a comparable pattern, with relatively high Jaccard similarity at 40% lung cancer cases that dropped as class imbalance increased, reaching its lowest values for models trained on the original imbalanced distribution. Rank agreement followed similar patterns, decreasing with greater imbalance and falling below 10% across all models when compared against the balanced reference model (Figure 4b).

To formally assess whether explanation consistency differed between balanced and imbalanced models, we performed pairwise statistical testing. For each imbalanced model and explanation technique, we generated explanations for 100 test instances and computed the Jaccard similarity and Rank Agreement between the top-10 feature rankings of the imbalanced model and its balanced reference. This produced 100 paired values per metric for each setting. We then used the Wilcoxon signed-rank test to evaluate whether these distributions were significantly different from the balanced reference. For both LIME and SHAP, all models trained on imbalanced datasets showed significant differences in both Jaccard similarity and Rank Agreement (*p* < 0.01), indicating that their feature rankings substantially differed from their respective balanced reference model.

Therefore, the consistency of LIME and SHAP explanations decreased with increasing class imbalance when evaluated against a balanced dataset, highlighting that both techniques were sensitive to changes in class distributions. The models trained on more balanced data generally performed better compared to imbalanced datasets in terms of AUC, sensitivity, and specificity; however, the relationship between predictive performance and explanation consistency is not perfectly linear across all models and class imbalance levels. This suggests that although poorer model performances often coincide with less reliable feature attribution rankings, factors related to class distribution also influence explanation consistency. Therefore, it is important to carefully evaluate explanation techniques in imbalanced clinical datasets because changes in data balance can affect both model performance and interpretability.

#### 3.2.2 Evaluation of class-wise explanation consistency

**Figure 5:**
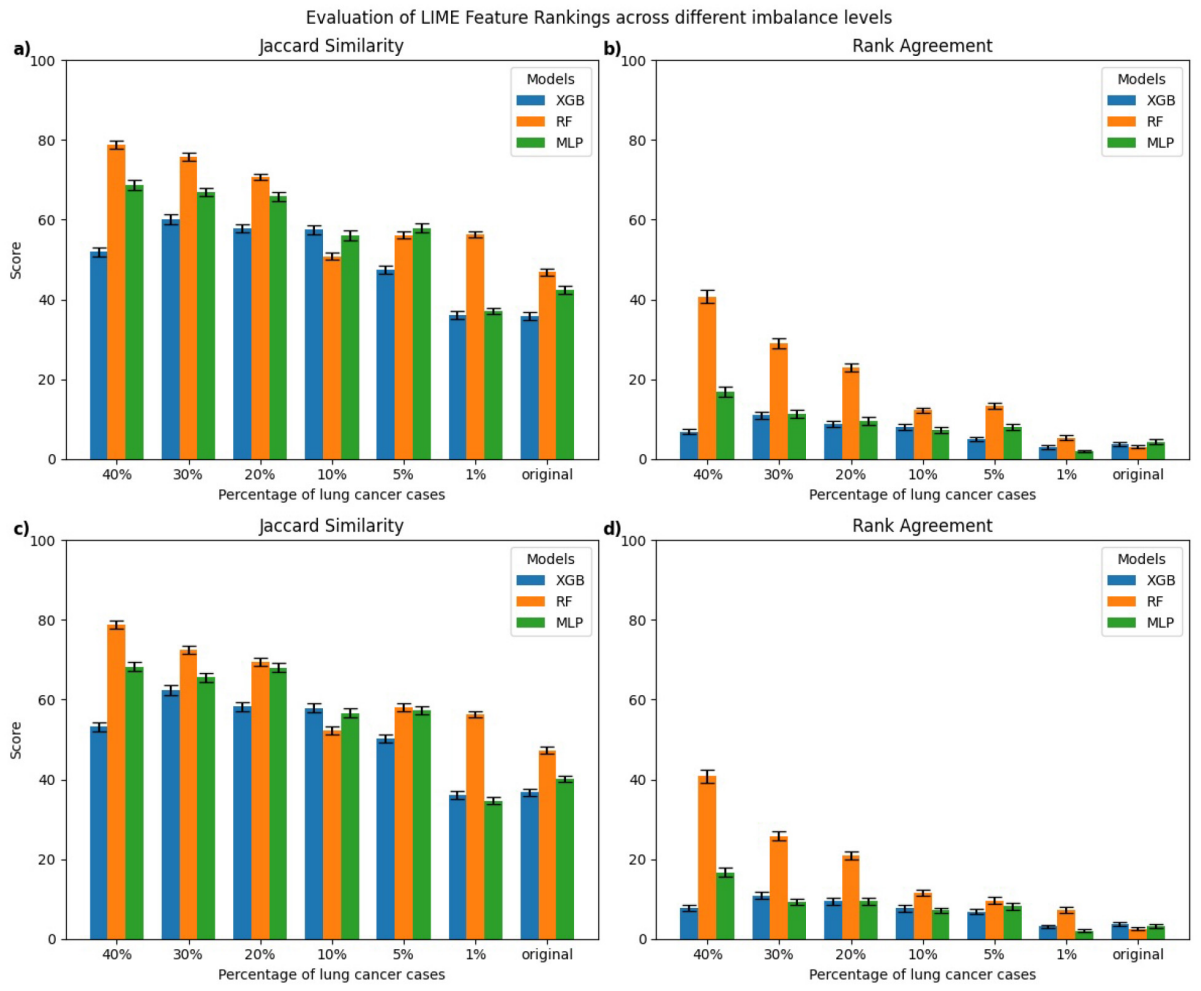
Class-wise comparison of the top-10 feature rankings generated by LIME across different imbalanced models relative to the balanced model using: **(a)** Jaccard Similarity Index for explanations of lung cancer cases, **(b)** Rank Agreement for lung cancer cases, **(c)** Jaccard Similarity Index for non-lung cancer cases, **(d)** Rank Agreement for non-lung cancer cases.

**Figure 6:**
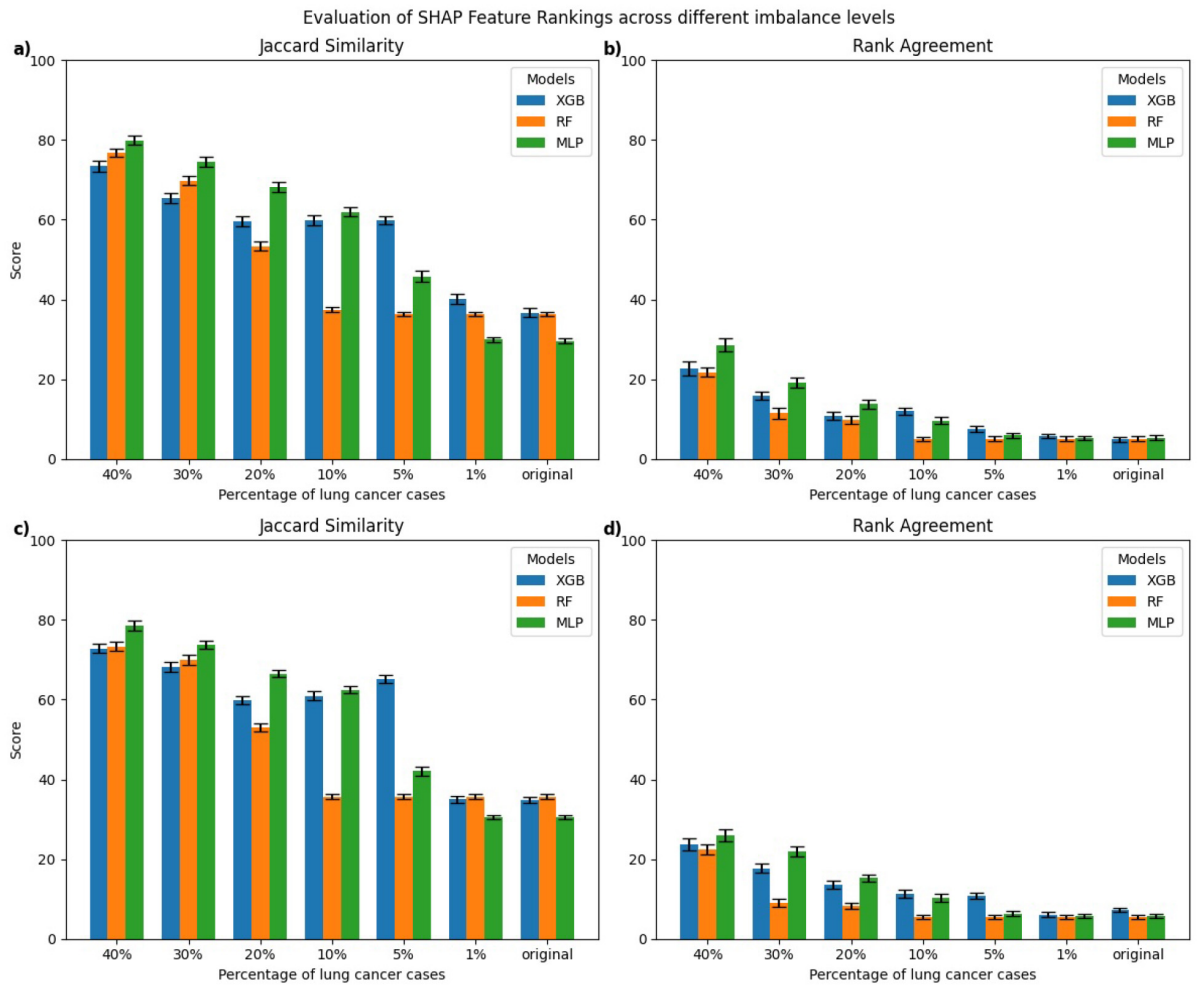
Class-wise comparison of the top-10 feature rankings generated by SHAP across different imbalanced models relative to the balanced model using: **(a)** Jaccard Similarity Index for explanations of lung cancer cases, **(b)** Rank Agreement for lung cancer cases, **(c)** Jaccard Similarity Index for non-lung cancer cases, **(d)** Rank Agreement for non-lung cancer cases.

In this section, we present a class-wise evaluation of explanation consistency for the minority (lung cancer cases) and majority (non-lung cancer cases) classes using LIME and SHAP. Figures 5, 6 show class-wise comparisons of the top-10 feature rankings from balanced and imbalanced models, evaluated using the Jaccard similarity index and Rank Agreement for LIME and SHAP, respectively.

For LIME, explanations of lung cancer cases (Figures 5a, b) showed the highest Jaccard similarity in RF models trained on 40% lung cancer cases, followed by a gradual decline as class imbalance increased. XGB models displayed a similar pattern, with relatively higher Jaccard similarity at a higher percentage of lung cancer cases and the lowest similarity when trained on the original imbalanced dataset. Comparable results were observed for explanations of non-lung cancer cases (Figures 5c, d), where Jaccard similarity was consistently lowest for models trained on the original imbalanced data. Rank Agreement followed a similar trend across both classes, with agreement against the balanced reference models decreasing as the imbalance increased, showing minimal overlap in feature orderings for the most imbalanced models.

For SHAP, explanations of lung cancer cases (Figures 6a, b) showed that MLP models trained on 5%–40% lung cancer cases achieved higher Jaccard similarity than XGB and RF models trained on the same distributions. However, as the imbalance increased further, their similarity declined below that of RF and XGB. A similar pattern was observed for explanations of non-lung cancer cases (Figures 6c, d). Rank Agreement also decreased steadily with greater imbalance for both classes, with all models sharing less than 5% of feature orderings with their balanced reference when trained on the original imbalanced dataset.

To formally assess whether explanation consistency differed between balanced and imbalanced models, we performed statistical testing for explanations of both minority and majority classes, following the procedure described in Section 3.2. For both LIME and SHAP, explanations of minority and majority classes showed significant differences in Jaccard similarity and Rank Agreement when comparing imbalanced models with their balanced reference (*p* < 0.01), indicating that feature rankings were substantially altered under imbalance. We also tested whether explanation consistency differed significantly between minority and majority classes within each imbalanced model. For this, we compared the distributions of Jaccard similarity and Rank Agreement of each imbalanced model relative to their balanced reference for the two classes. No significant differences were observed (all *p* > 0.01). This suggests that imbalance primarily affects Jaccard similarity and Rank Agreement of both classes relative to the balanced reference but does not create a significant difference between classes within the same imbalanced model.

#### 3.2.3 An empirical illustration of variation in explanations by LIME and SHAP

We present an empirical example demonstrating how LIME and SHAP explanations differ when models are trained on balanced vs. imbalanced data.

**Figure 7:**
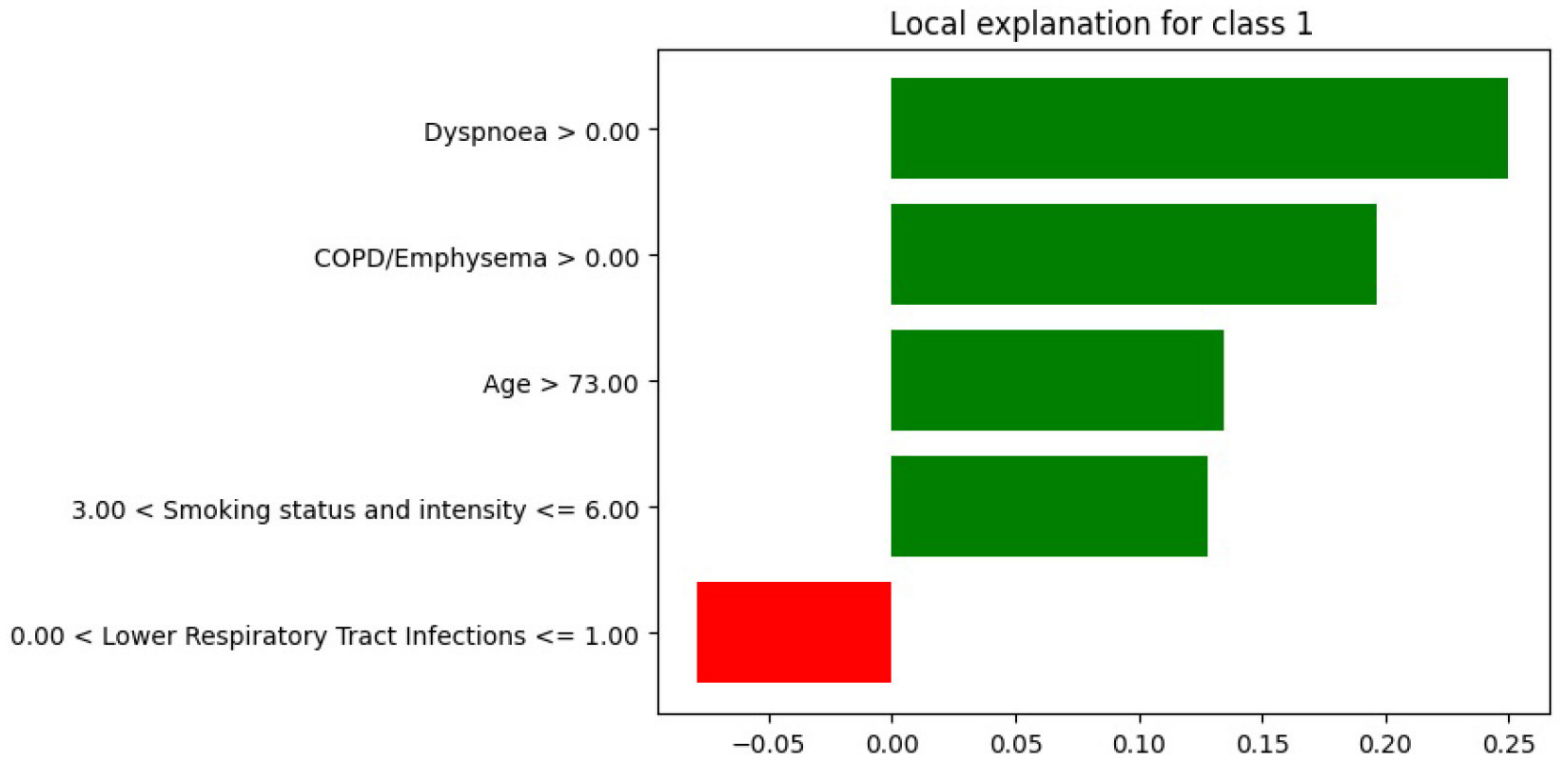
LIME explanations from the XGB model trained on balanced data for a patient diagnosed with lung cancer.

**Figure 8:**
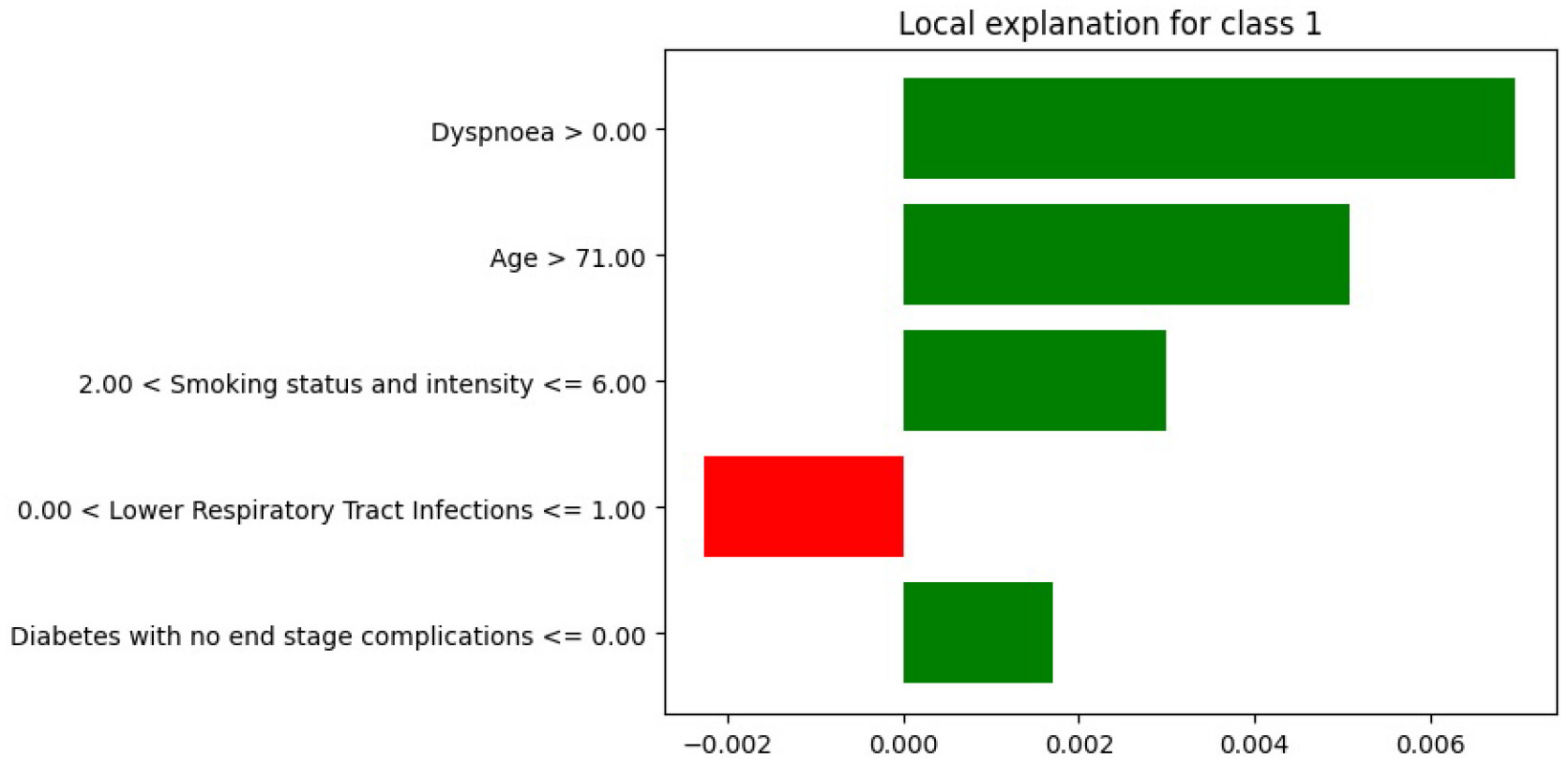
LIME explanations from XGB model trained on original imbalanced data for a patient diagnosed with lung cancer.

We selected a patient diagnosed with lung cancer and examined the LIME coefficients, that is, the weights of the local surrogate linear model for this individual prediction. Figures 7, 8 display the LIME explanations generated from the XGB model trained on balanced and original imbalanced datasets for the same patient, respectively. Each bar in the plots represents the contribution of a feature to the predicted outcome for the selected patient, with feature values shown alongside.

In Figure 7, the feature *age* is assigned a coefficient of 0.15. In contrast, in Figure 8, the same feature receives a coefficient of only 0.01 when the model is trained on the original imbalanced dataset. This substantial difference in attribution highlights how the LIME explanations and thus the local interpretability are affected by the underlying training distribution of the model. Moreover, we see that the feature *diabetes with no end stage complications* appears in the top-5 important features for the patient when the model is trained on the original imbalanced data (Figure 8) but does not appear for the balanced model. These findings reinforce the theoretical result that changes in the training data distribution lead to changes in the learned model *f*, which in turn alters the predictions on locally perturbed samples and thus results in different LIME surrogate models. Therefore, LIME feature attributions are not consistent across models trained with differing class distributions.

**Figure 9:**
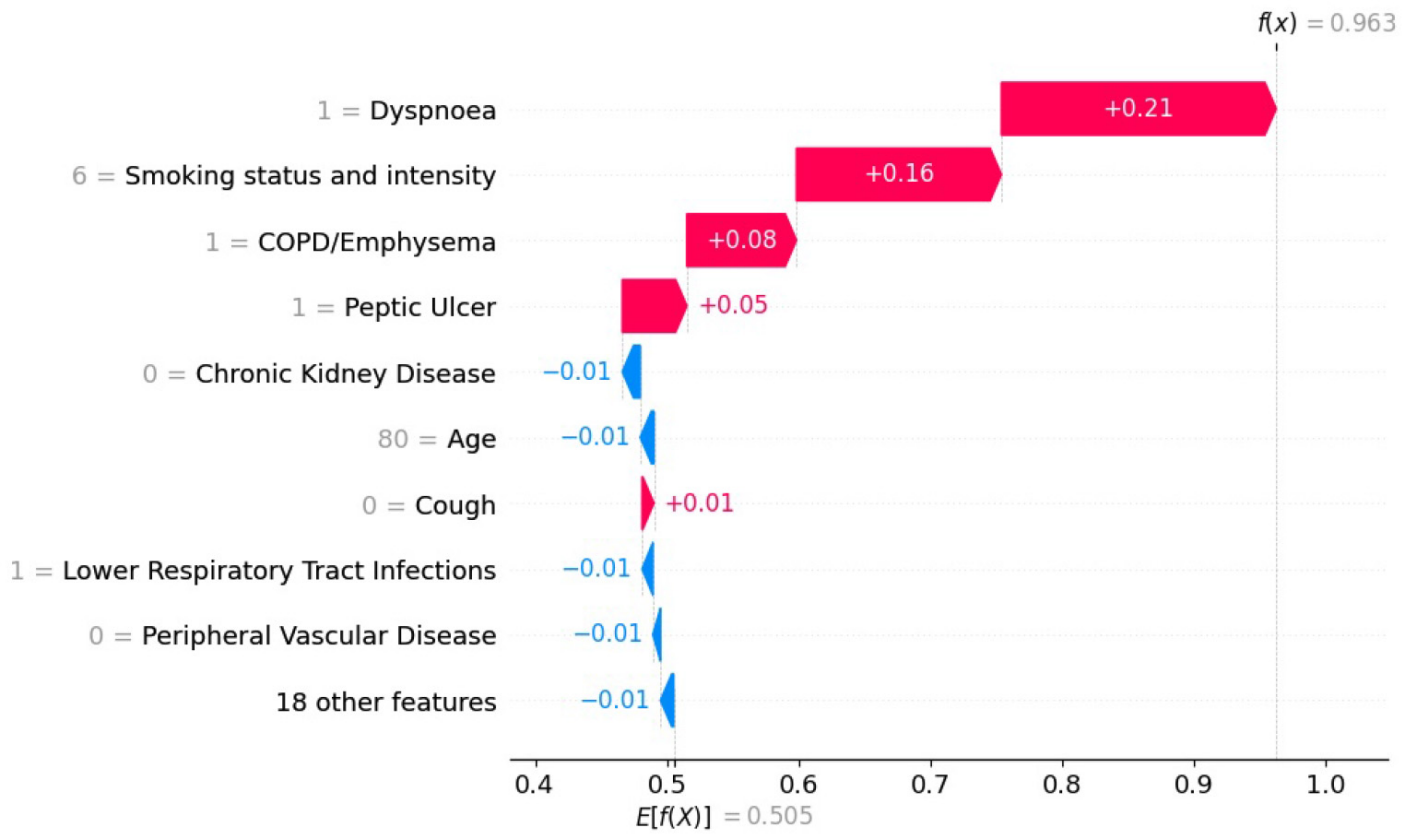
SHAP values with XGB model trained on balanced data for patient diagnosed with lung cancer.

**Figure 10:**
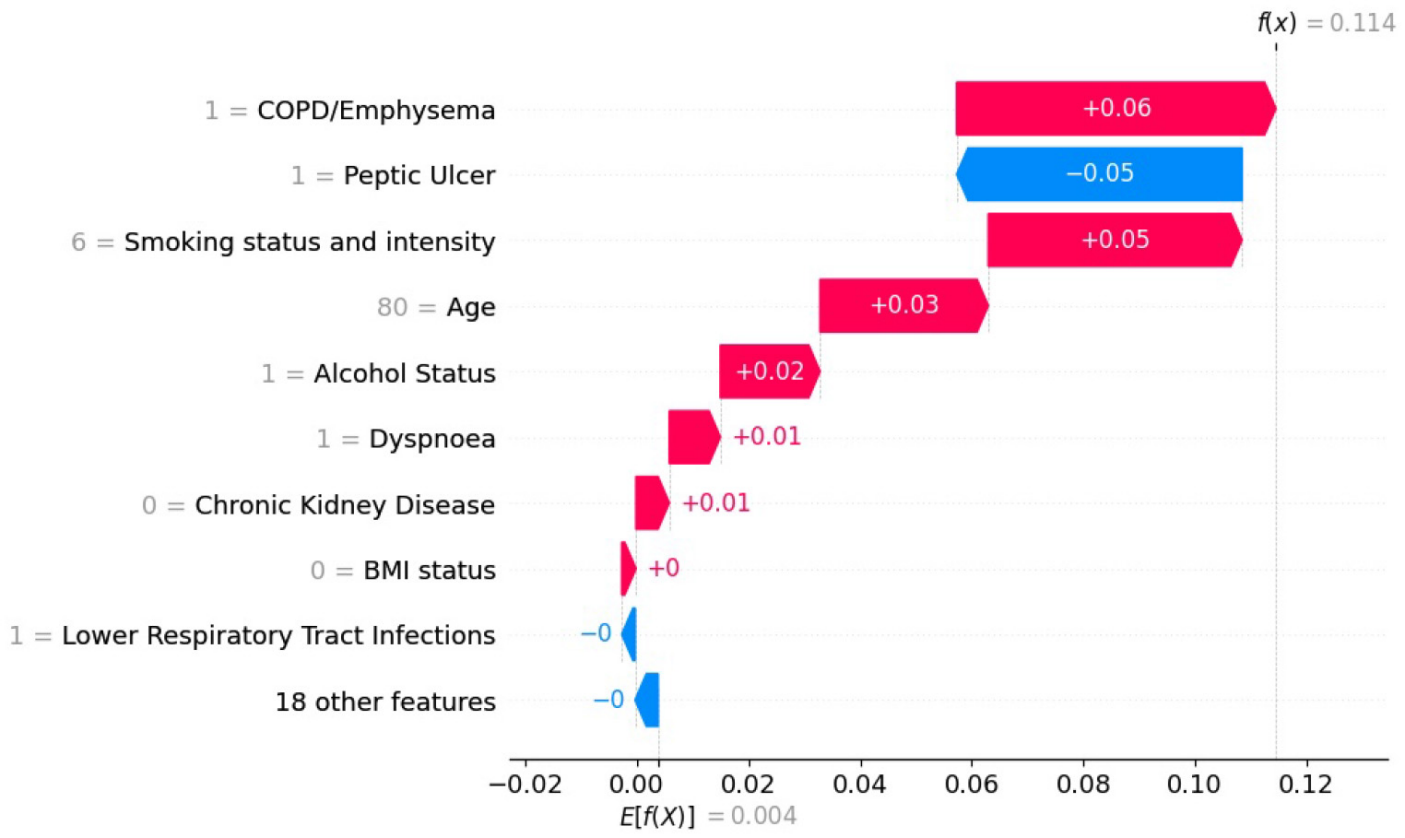
SHAP values with XGB model trained on original imbalanced data for a patient diagnosed with lung cancer.

Similarly, we illustrate how SHAP values differ for the same patient as above when using an XGB model trained on the original imbalanced dataset compared to the balanced dataset. For this instance *x*^(*j*)^, we compute the SHAP value $\phi_{j}\left(f,x^{\left(j\right)}\right)$ for each feature *i*. Figures 9, 10 show SHAP waterfall plots for this patient, using models trained on the balanced and the original imbalanced dataset, respectively. The *x*-axis represents the SHAP values, and the *y*-axis represents the individual features, along with their specific values for the selected patient.

In Figures 9, 10 the top of each plot shows the predicted probability *f*(*x*^(*j*)^) for the selected patient and the model's expected output *E*[*f*(*X*)] along the *x*-axis. For this positive case, the model trained on imbalanced data produces a lower predicted probability because it is biased toward the majority class (Figure 10). Since SHAP values are additive, their sum equals the difference between the predicted and expected probabilities. A lower predicted probability, therefore, reduces the absolute size of the SHAP values, as seen in Figure 10, where the SHAP values of the top-10 features are smaller than in Figure 9. For example, in Figure 9, the balanced model assigns a SHAP value of 0.16 to feature *smoking status and intensity*, making it the second most important feature. In the imbalanced model (Figure 9), the same feature for the same patient has a SHAP value of 0.05 and is ranked third. This shows that class imbalance affects both the model's predictions and the feature attribution values used for interpretation, supporting our theoretical explanation in Section 2.6.

### 3.3 Evaluation of PDPs under class imbalance

To further evaluate the changes in model behavior due to class imbalance, PDPs were generated for the top-2 clinically plausible features, *age* and *smoking status and intensity* (Callender et al., 2023; Rai et al., 2024) for models trained on balanced and the original imbalanced datasets.

**Figure 11:**
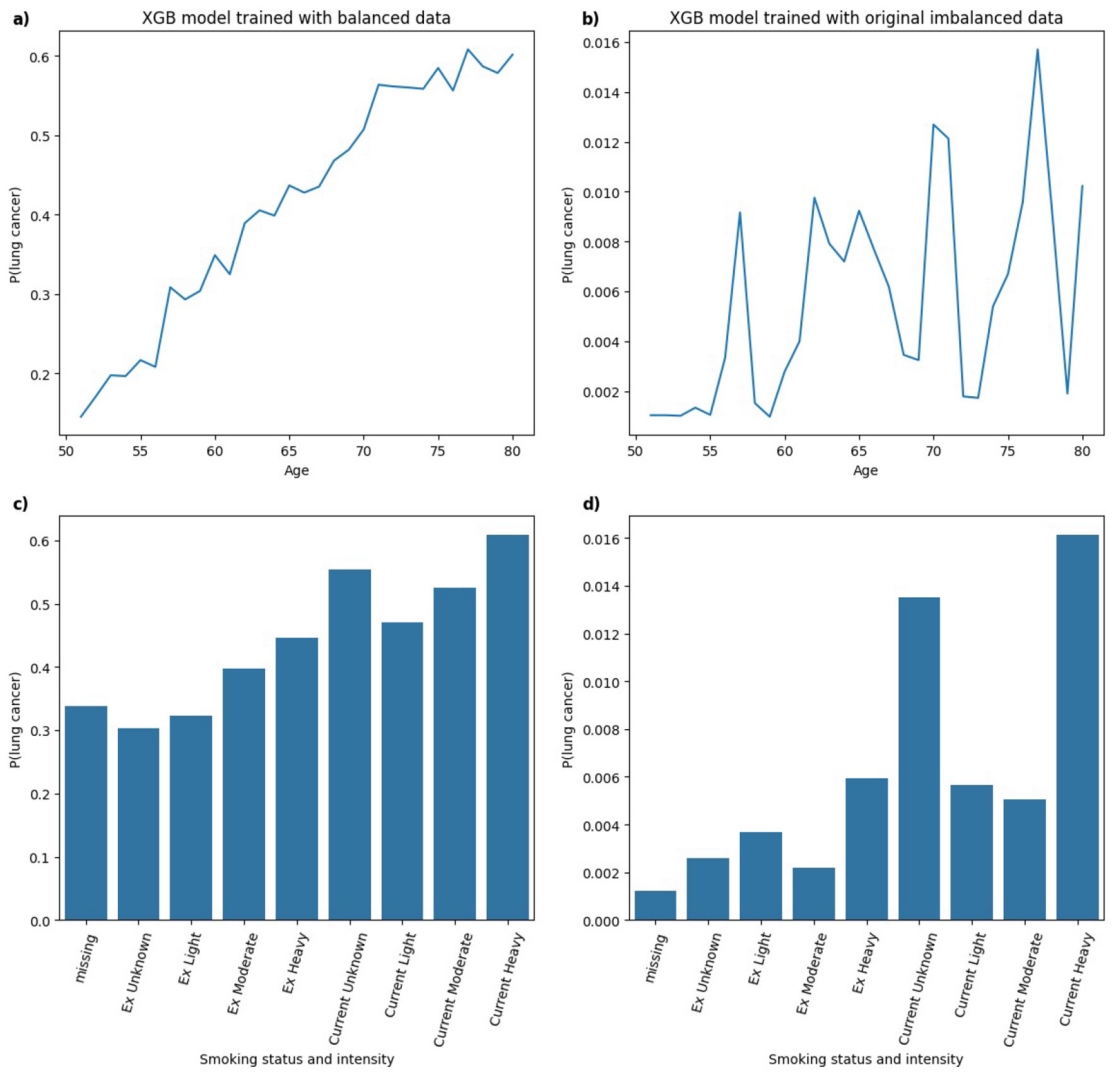
Comparison of PDPs for **(a, b)** *age* and **(c, d)** *smoking status and intensity* in XGB models, trained on balanced **(a, c)** and imbalanced **(b, d)** data, showing changes in model behavior.

Figure 11 illustrates the PDPs for the predictors *age* and *smoking status and intensity* in XGB models trained on balanced and highly imbalanced datasets. For the balanced model, a monotonic relationship is observed between *age* and predicted probability of lung cancer diagnosis, that is, the likelihood increases with age. This trend aligns with epidemiological evidence reported in Cancer Research UK (2019), which shows that lung cancer incidence rates rise substantially between ages 40 and 79. In contrast, the model trained on the original imbalanced dataset displays a non-monotonic, erratic relationship between age and lung cancer probability, suggesting that the model fails to learn a clinically coherent pattern under severe class imbalance. Similarly, for *smoking status and intensity*, the model trained on balanced data assigns increasing probabilities of lung cancer from *Ex Unknown* to *Current Unknown* smokers, with the highest for *Current Heavy* smokers. This pattern is consistent with findings from (Liao et al. 2023) where heavy smoking is associated with highest hazard ratio relative to other categories. However, the imbalanced model's PDP for *smoking status and intensity* shows less consistent structure. While *Current Unknown* and *Current Heavy* smokers still yield higher predicted probabilities, it lacks the smooth or clinically intuitive pattern observed in the balanced model.

**Figure 12:**
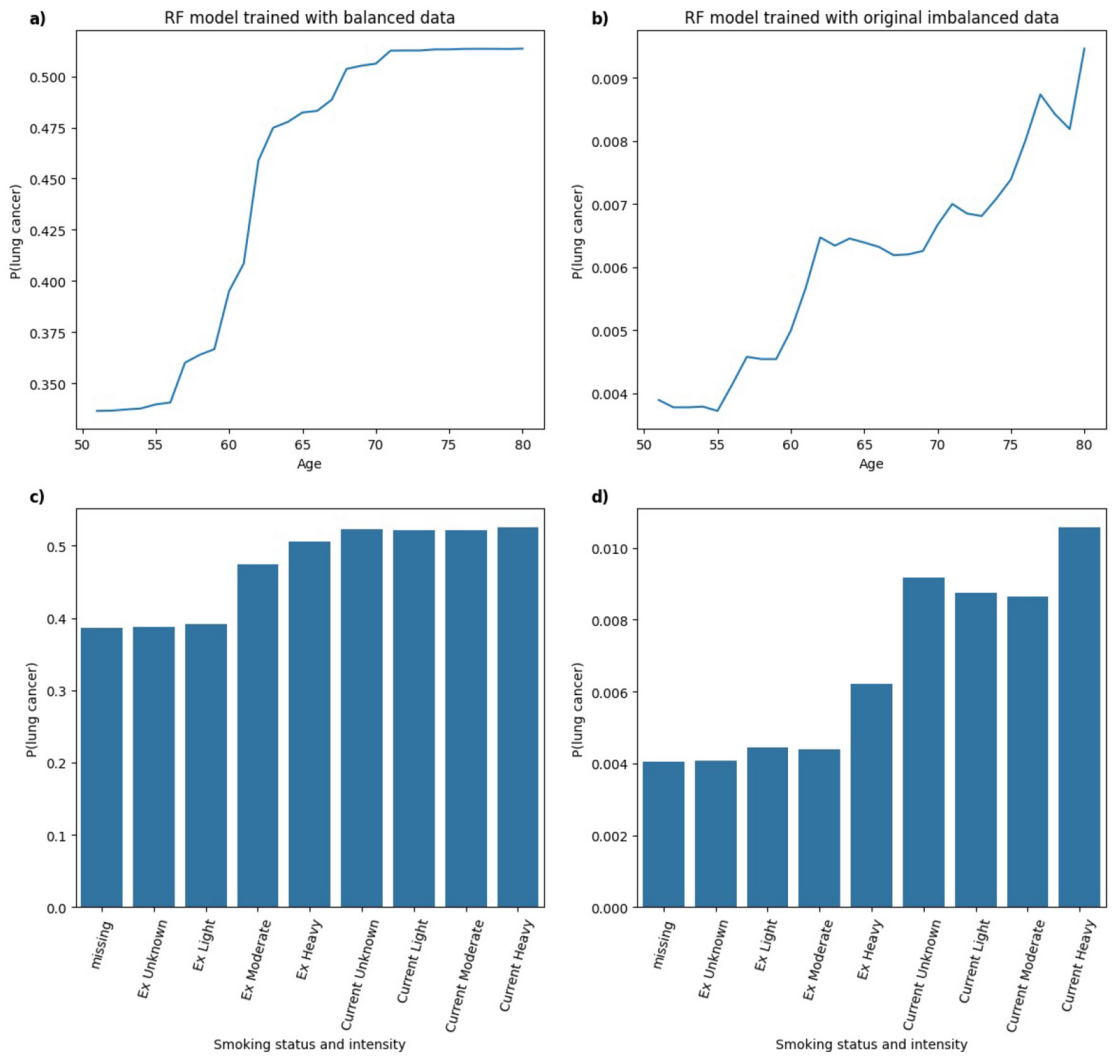
Comparison of PDPs for **(a, b)** *age* and **(c, d)** *smoking status and intensity* in RF models, trained on balanced **(a, c)** and imbalanced **(b, d)** data, showing changes in model behavior.

In Figure 12, for the RF model trained on balanced data, the predicted probability of lung cancer shows an upward trend with *age*, leveling off between ages 70 and 80. This indicates a strong, clinically plausible relationship between increasing age and lung cancer risk. In the RF model trained on an imbalanced dataset, a generally monotonic relationship with age is still present, but the pattern is less distinct compared to the balanced model, suggesting that class imbalance may reduce the model's ability to learn specific patterns. With respect to *smoking status and intensity*, the balanced RF model shows higher predicted probabilities for Current and Ex smokers, although the variation across smoking intensities is less pronounced than observed in the XGB model. In the RF model trained on imbalanced data, the predicted probabilities follow a pattern similar to the balanced model with higher lung cancer risk associated with *Current* smokers compared to *Ex* smokers. In this instance, the model captures clinically relevant distinctions in smoking behavior in both balanced and imbalanced data.

**Figure 13:**
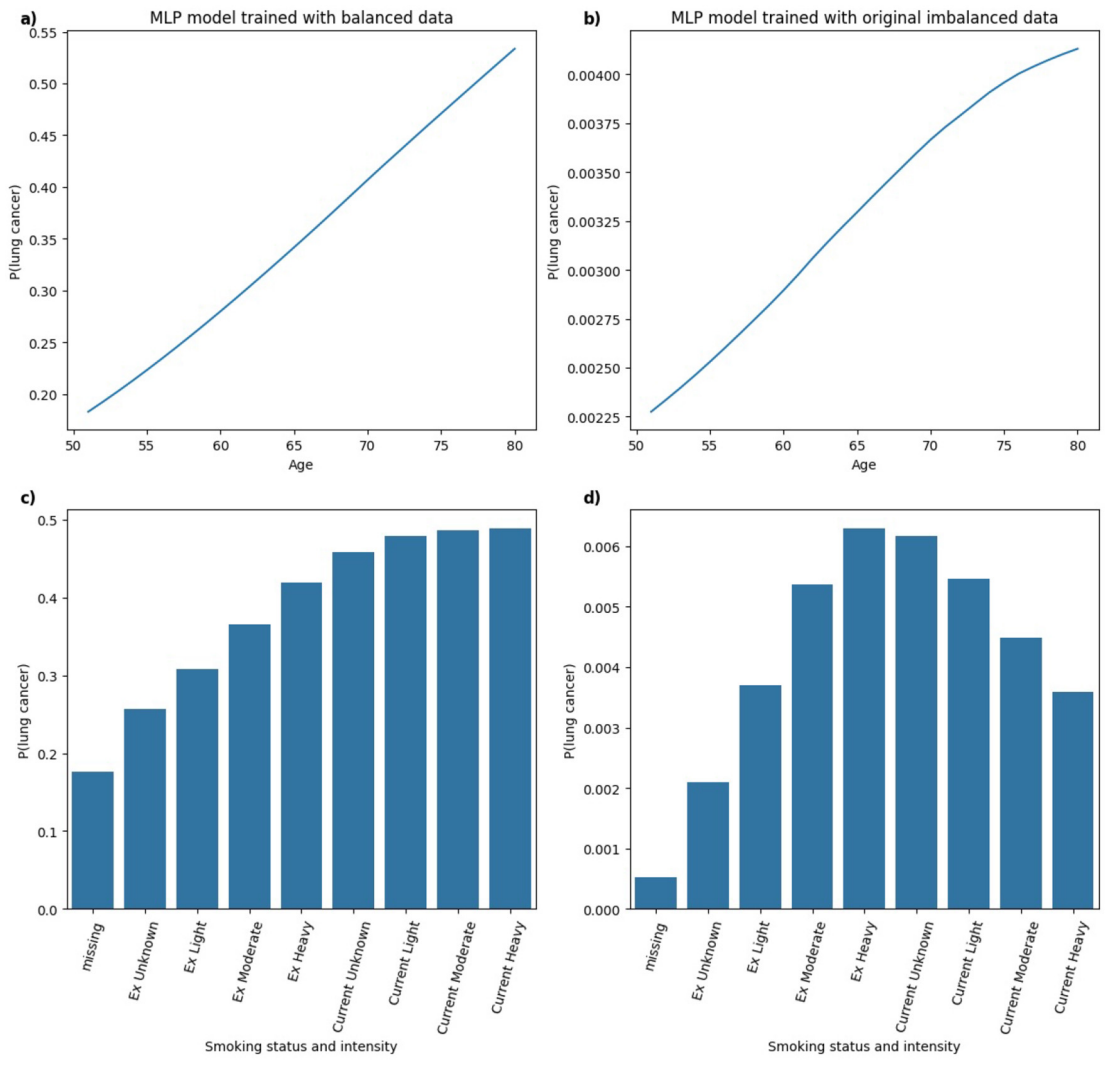
Comparison of PDPs for **(a, b)** *age* and **(c, d)** *smoking status and intensity* in MLP models, trained on balanced **(a, c)** and imbalanced **(b, d)** data, showing changes in model behavior.

In Figure 13, for the MLP model trained on both balanced and imbalanced datasets, the relationship between *age* and the predicted probability of lung cancer appears approximately linear. In the balanced model, there is a clear and clinically coherent pattern that the probability of lung cancer increases with *smoking status and intensity*, with *Current* smokers exhibiting higher predicted risk than *Ex* smokers, aligning well with established findings in the literature. In contrast, for the MLP model trained on the original imbalanced dataset, the pattern deviates from this expected trend, resembling a bell-shaped curve. The highest predicted probability is assigned to *Ex Heavy* smokers, suggesting that the model may misrepresent the true risk distribution under severe class imbalance.

The analysis of PDPs across models trained on balanced and original imbalanced datasets suggests that class imbalance may degrade the interpretability or clinical relevance of model behavior. For some models (e.g. RF), predictions remained relatively consistent across models trained under different class distributions, capturing clinically plausible relationships in both balanced and imbalanced scenarios. However, other models (e.g. MLP) showed notable shifts in learned patterns under class imbalance, particularly in interpreting *smoking status and intensity*. Hence, model-specific evaluation of explanation robustness and consistency is essential in high-stakes settings such as healthcare, where trust in both model predictions and explanations is critical.

## 4 Discussion

Despite the rapid advancement and widespread publication of AI techniques in medical research, statistical methods remain the primary tools used in clinical practice. For example, QRISK3 (Hippisley-Cox et al., 2017), a Cox proportional hazards model used to estimate 10-year cardiovascular risk, is routinely implemented in UK primary care by the National Health Service (NHS). These models are favored for their interpretability, as hazard ratios provide a clear and direct way to compare risks across patient groups. Similarly, logistic regression's output in the form of odds ratios is widely understood and trusted by clinicians. The Liverpool Lung Project (LLP) risk model (Cassidy et al., 2008), which applies logistic regression to case-control data, has been used in selecting individuals for the UK Lung Cancer Screening Trial (Field et al., 2016). While ML models such as XGB and RF (Pan et al., 2023; Rai et al., 2023) have demonstrated improvements in discrimination and calibration over traditional statistical models, their adoption in clinical practice remains limited. This is especially true for risk prediction models using structured tabular data, where statistical models still perform quite well and are inherently interpretable.

To facilitate the clinical acceptance of ML-based decision support tools, XAI techniques must be stable, trustworthy, and robust, especially when dealing with rare health outcomes such as lung cancer, where class imbalance is common. In this study, we have shown that ML models trained on imbalanced data can produce different explanations than those trained on balanced data, which not only affects predictive performance but also undermines the reliability of XAI techniques. Previous work, such as (Chen et al. 2024), has investigated how class imbalance affects the stability of LIME and SHAP explanations by measuring variance across multiple runs. However, their study focuses on a financial credit dataset. Our study extends this by assessing consistency in feature attribution rankings across models trained under different class distributions by using models trained on balanced data as a reference. We also examined how explanation consistency relates to model performance and provided theoretical foundations to explain why LIME and SHAP explanations become less consistent under skewed class distributions. Moreover, while (Wang et al. 2023) uses ML models with SHAP and PDP to interpret their models to predict Chronic Obstructive Pulmonary Disease (COPD), their study employed SMOTE to address an imbalance ratio of approximately 5%. Our findings show that PDPs are sensitive to the training data distribution, which implies that different levels of class imbalance can lead to varying model interpretations, highlighting the need for careful treatment of class imbalance when using visual interpretability tools. While (Lai et al. 2019) explores feature importance variability across models using textual data, they do not provide theoretical explanations for the observed inconsistencies. In contrast, our study contributes theoretical insight into how and why LIME and SHAP explanations change under different class distributions, which is also supported by empirical evidence.

This study has several limitations. The reference dataset used for comparison was created through an undersampling approach to achieve class balance. Future research could explore whether similar findings hold when using oversampling methods such as SMOTE. However, the balanced reference dataset created by random undersampling may introduce biases. By artificially reducing the number of non-lung cancer cases, the models are trained on a distribution that does not reflect real-world prevalence, which can affect model calibration (Piccininni et al., 2024). In particular, the estimated probabilities of lung cancer are inflated under undersampling even though class balance may improve model performance. Future study should therefore explore alternative balancing strategies such as oversampling (e.g., SMOTE) or matching-based approaches and systematically assess how different methods affect both calibration and explanation consistency. Additionally, our experiments were conducted exclusively on a lung cancer cohort derived from the CPRD dataset. Future studies could involve replicating the analysis across diverse datasets to strengthen the generalisability of our conclusions. Moreover, our experiments were conducted on a lung cancer cohort from the CPRD dataset (version 2021), which included follow-up data up to 2020 and was the most recent version available to us. Future study could extend our analysis to more recent releases to confirm whether the findings hold in updated populations. Another limitation is the lack of external validation to evaluate model performance and explanation consistency in an independent dataset. Such validation will be essential to establish robustness and clinical applicability. However, we argue that the theoretical foundations we provide for LIME and SHAP offer a rationale for why explanation consistency deteriorates under class imbalance, supported by our empirical evaluation with the CPRD dataset. These insights are further supported by the patterns observed in PDPs.

Hence, a systematic framework for evaluating the reliability and consistency of XAI techniques is needed before these methods can be deployed in healthcare settings. Therefore, more studies on evaluating XAI techniques are needed to build clinical trust and enable the broader adoption of AI in primary care.

## 5 Conclusion

In this study, we developed a comparative framework using Jaccard similarity index and Rank Agreement to evaluate the consistency of LIME and SHAP explanations under class imbalance in predicting lung cancer risk from CPRD data. A balanced dataset achieved using random undersampling with an equal number of lung cancer and non-lung cancer cases is used as a reference to compare explanation consistency across models trained under different class distributions. We trained XGB, RF, and MLP models and examined the relationship between model performance and explanation consistency to identify potential trade-offs between performance and interpretability. Furthermore, we conducted a deeper theoretical and empirical investigation of how the consistency of explanations based on LIME and SHAP varies under class imbalance using Jaccard similarity and Rank Agreement, and how this impacts interpretability. We further conducted a detailed assessment of explanation consistency for the minority (lung cancer cases) and majority (non-lung cancer cases) classes. Lastly, we incorporated PDPs into our evaluation framework to assess how the model's learning of clinically relevant features for predicting lung cancer risk changed across balanced and imbalanced datasets.

First, we find that the AUC of the models decreased as class imbalance increased, especially for MLP and XGB models, where performance dropped significantly when trained on datasets with 1% class imbalance and the original lung cancer prevalence. RF achieved the highest sensitivity when trained with 40% lung cancer cases, while both XGB and MLP maintained a good trade-off between sensitivity and specificity across all imbalance levels. Second, the consistency of LIME and SHAP explanations decreased as class imbalance increased when evaluated against a balanced dataset, highlighting that both techniques were sensitive to changes in class distribution. This effect was also observed when looking specifically into explanations of lung cancer and non-lung cancer cases, confirming that explanation consistency deteriorates equally for minority and majority classes under class imbalance. Third, we analyzed the internal mechanisms of LIME and SHAP and empirically demonstrated that LIME coefficients and SHAP values shrink under models trained on a highly imbalanced dataset compared to a balanced dataset. Lastly, PDPs revealed changes in model behavior between balanced and imbalanced training scenarios with respect to clinically relevant features for predicting lung cancer risk: *age* and *smoking status and intensity*. Our findings suggest that model interpretability, rather than just predictive performance, is affected by class imbalance. Therefore, researchers and practitioners must exercise caution when applying resampling techniques or interpreting XAI outputs, particularly in imbalanced data scenarios, which are common in healthcare data.

Our findings have important clinical implications. In settings where models are trained on highly imbalanced data, explanation variability may lead to different clinical features being highlighted as important, undermining clinical trust and affecting decision-making. To mitigate this, we recommend using balanced models as reference baselines when evaluating explanation consistency. In practice, explanations of ML models should be benchmarked across multiple XAI techniques and under different training distributions to identify features that are consistently important. Future research should also focus on developing explanation techniques that are inherently more robust to class imbalance. Establishing a systematic framework for evaluating explanation consistency under varying data distributions will be an important step toward the reliable and clinically responsible use of XAI in healthcare.

## Data Availability

The data analyzed in this study is subject to the following licenses/restrictions. Data used in this work is available through CPRD and is not publicly available. Requests to access these datasets should be directed to https://www.cprd.com/data-access.
